# Typhoid fever in paediatric patients in Quetta, Balochistan, Pakistan

**DOI:** 10.12669/pjms.294.3251

**Published:** 2013

**Authors:** Muhammad Naeem Khan, Muhammad Shafee, Kamran Hussain, Abdul Samad, Muhammad Arif Awan, Abdul Manan, Abdul Wadood

**Affiliations:** 1 Muhammad Naeem Khan, Department of Microbiology, University of Balochistan, Quetta, Pakistan.; 2 Muhammad Shafee, Lecturer, Center for Advanced studies in Vaccinology & Biotechnology (CASVAB), University of Balochistan, Quetta, Pakistan.; 3 Kamran Hussain, Microbiologist Children Hospital, Quetta, Pakistan. Center for Advanced studies in Vaccinology & Biotechnology (CASVAB), University of Balochistan, Quetta, Pakistan.; 4 Abdul Samad, Assistant Professor, Center for Advanced studies in Vaccinology & Biotechnology (CASVAB), University of Balochistan, Quetta, Pakistan.; 5 Muhammad Arif Awan,Assistant Professor, Center for Advanced studies in Vaccinology & Biotechnology (CASVAB), University of Balochistan, Quetta, Pakistan.; 6 Abdul Manan, Lecturer, Department of Microbiology, University of Balochistan, Quetta, Pakistan.; 7 Abdul Wadood, Chairman, Department of Microbiology, University of Balochistan, Quetta, Pakistan.

**Keywords:** Prevalence, Typhoid Fever, Typhidot, Widal

## Abstract

***Objectives: ***To determine the seropositivity of typhoid fever in febrile pediatric patients presenting to tertiary care center.

***Methods:*** This observational study was conducted at Children Hospital Quetta (CHQ) from July 2011 to March 2012. The children with three or more days fever, no obvious focus of infection and clinically suspected of typhoid fever were screened. Sterile Blood samples were obtained from febrile patients and Widal and Typhidot® tests were performed for the diagnosis of Typhoid fever in the suspected populations.

***Results:*** Total of 2964 clinically suspected patients were screened for typhoid fever. Of these, 550 (18.6%) patients were positive serologically. The higher prevalence of the disease in hot summer season and increasing pattern of the disease was observed in summer days. The disease was higher in school age children under 5-10 years. Although non-significant association was observed on sex basis.

***Conclusion:*** The findings highlight the considerable burden of typhoid fever in pre-school and school-aged children. The variation in the disease pattern has also been observed under seasonal variation and different age groups, all of which need to be considered in deliberations to control the typhoid fever.

## INTRODUCTION

Enteric fever continues to be endemic in economically poor countries, although it has been eradicated from economically stable and developed nations by well-organized sanitation and hygienic water supply.^1^ It is the disease from prehistoric dates that has afflicted human populations due to contaminated water and food supplies.^2^ In the industrial world the realization of fecal contamination of food and water as the main source of transmission of the disease, helped to control and prevent typhoid fever in areas where it is now curtailed to local epidemics.^3^ The disease is endemic in the Indian sub-continent, South-East Asia, Africa, the Middle-East, South and Central America, where provision of pure water supplies and sewage control are inadequate.^4^ The Centre of Disease Control (CDC) reported that the incidence of typhoid fever in US citizens visiting to the Indian subcontinent was at least eighteen times higher than other geographical region of the world.^5^

Typhoid fever is caused by (*S.typhi*). The bacterium is transmitted by fecal-oral route, through contaminated water or food source. *S. typhi* is a multi-organ pathogen that inhabits the lymphatic tissues of the small intestine, liver, spleen and bloodstream of infected humans.^6^ Enteric fever is reported more frequently in children above five years of age having complications in more than one third of the patients.^7^ It was estimated that incidence among children aged 2–5 years was 573.2, 340.1 and 148.7 per 100,000 person per years in Pakistan, India and Indonesia, respectively. It was 451.7 cases per 100,000 persons per years among 2–15 year-olds in Pakistan. The rates were significantly higher in the south Asian countries (Pakistan and India) than in the south-east and north-east Asian countries (Viet Nam, Indonesia and China).^8^

It represents the fourth most common cause of death in Pakistan irrespective of the age.^9^ It may be a consequence of rapidly developing drug resistance, for instance 69% of *S. Typhi* isolates from blood were found multi drug resistant in Quetta, Pakistan.^10^

Although population-based data from Pakistan are inadequate, several hospital-based studies from different parts of the country have consistently shown a very high incidence of typhoid fever, especially in the younger age groups.^11^ Information from Delhi has indicated that the relative incidence of typhoid fever is considerably greater in preschool children.^12^ This study was designed to estimate the prevalence and seasonal variation of pediatric typhoid fever in Quetta, Balochistan, Pakistan.

## METHODS

***Study Area: ***Quetta is the largest city and the provincial capital of the Balochistan Province located at 30°12′38″N 67°1′8″E with an average elevation of 1,680 meters above sea level, making it Pakistan's only high-altitude major city. The present study was designed to estimate the prevalence and seasonal variation of pediatric typhoid fever. The study was started in July 2011 and continued until March 2012 at the Children Hospital Quetta (CHQ) under the supervision of medical experts.

***Patients inclusion criteria: ***Patients with fever over 72 hours, no obvious focus of infection and clinically suspected for typhoid fever (high fever, malaise, headache, constipation or diarrhoea) were included. Children 0-18 Years, suspected for typhoid fever by the physicians of CHQ were recruited in the study. Suspects were divided into different age groups, infants 0-1 year, pre-school children aged >1—5 years, school children aged >5—10 and >10—15 years, and adolescents above >15 years.

***Samples Collection: ***Three (3) ml of blood samples were withdrawn through aseptic venipuncture using a disposable syringe were transferred into sterile Gel Test tubes without any anticoagulant.

***Laboratory procedures: ***The serum was separated on centrifugation at 3500 rpm and was tested either by Widal or Typhidot®.

***Widal Test:*** The test was peformed as advised by manufacturers, briefly A drop of positive and negative control (Distilled water) was placed on clear plastic agglutination slide. Then one drop of the patient serum was placed on each of the four reaction circles. The Widal test antigen H was added to the both controls and a drop of O, H, AH and BH antigens were added to the remaining four circles already added with patient serum. The contents were mixed and rocked for one minute for visible agglutination reaction.

***Typhidot Test: ***The test was performed according to the manufacturer’s guidelines, briefly drop (30 ul) serum was added to the sample well ensuring no bubble formation. Then one drop of the buffer solution was added and the results were recorded with 15 minutes after wicking the sample.

## RESULTS

A total of 2946 patients clinically suspected for enteric fever were subjected to evaluation for typhoid fever using Widal (n=1526 Patients) and typhoid test (n=1420 patients). Five hundred and fifty patients were positive with overall prevalence of 18.66%. Two hundred sixty six (48.36%) were detected by using Typhidot whereas 284 (51.63%) by using Widal (Glass Slide Agglutination) test.

Out of 354 suspected patients, only 16 (4.5%) were positive in the age group 0-1 year whereas in 1-5 years age a total of 1261 patients were screened for typhoid fever and 216 were found positive with 17.12% prevalence. Similarly 822 patients of 5-10 years of age were subjected and 183 were found positive indicating 22.2% prevalence. While 401 patients of 10-15 years of age were screened and 108 were found positive with 26.9% prevalence. In Adolescent group, 108 patients were suspected and 27(25%) were positive.

**Fig.1:**
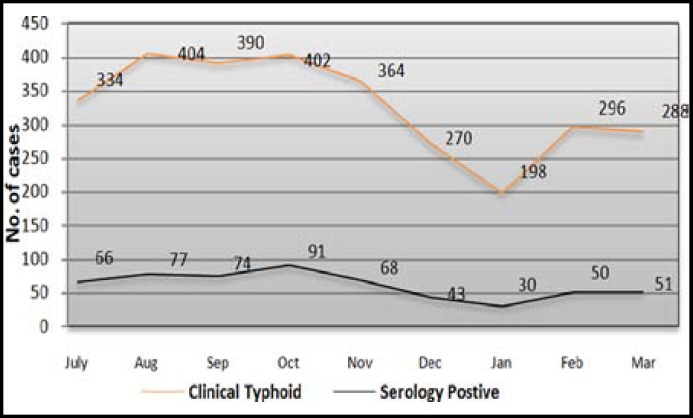
Graphical presentation of typhoid Cases in summer and winter season in Quetta

On sex basis Out of 1678 male patients 318 (18.95%) were positive while 232 Female patients were positive out of 1268 patients indicating 18.3% prevalence. The prevalence of typhoid varied seasonally, ranging from minimum 151.5 cases in the month of January to maximum 226.3 cases in October per 1000 suspected febrile episodes. The data collected revealed that higher prevalence during the summer (July-September) and autumn seasons (October–November) while lower frequency occurred during the winter season (December-February). The increasing pattern of the disease with increase in temperature was recorded (Fig.1).

**Table-I:** Age/sex wise prevalence of typhoid in patients visiting Children Hospital, Quetta

*Age groups (Years)*	*Clinical Subjects*	*Positive cases*	*Percentage (%) Positive*
0—1	354	16	4.51
>1—-5	1261	216	17.12
>5—10	822	183	22.26
>10—15	401	108	26.93
>15	108	27	25
Total	2946	550	18.66
Male	1678	318	18.95
Female	1268	232	18.30
Total	2946	550	18.66

## DISCUSSION

Typhoid is a serious public health problem in third world countries. South-East and South-Central Asian countries have the highest incidence (>100/100,000 cases/year).^13^ In Pakistan, typhoid fever is endemic, major factors for high prevalence rate include; overcrowding, illiteracy, poverty, poor sanitation and inadequate facilities for safe drinking water supply. A blood culture and serology based survey from Karachi, Pakistan revealed an incidence of 170 and 710 per 100,000 respectively.^14^

The collected data revealed less prevalence in preschool aged children (>1-5 years) than school aged children (10-15 years). These findings corroborate with Sinha et al, Lin et al and Rafiq et al who reported 44%, 11% and 17% typhoid cases in preschool ages (1-5 years) in India, Viet-Nam and Pakistan respectively.^12^^,^^15^^,^^16^ However, according to Levi et al and Ferric et al typhoid fever in children under five years is uncommon and subclinical.^17^^,^^18^ The apparent absence of culture-positive typhoid fever in children less than two years of age may be due to natural reluctance and difficulty to draw 5 ml blood from infants. Our study reflects high prevalence in preschool and adolescents, whereas Crump et al, reported heavy burden of typhoid fever in infants, children, and adolescents.^13^ High prevalence in school-aged children and adolescents may be attributed to poor hygienic conditions, eating and drinking outside the home from street vendors, consuming cold ice creams and beverages which are prepared under unhygienic conditions.

The maximum prevalence of typhoid fever was recorded in summer (June-October) while minimum in winter season (November-March). Total of 308 (n=1530) patients were found serologically positive with 20.13% prevalence in summer as compared to 17% in winter where 242 (n=1416) patients were found positive (Fig.1). In congruence with seasonality patterns elsewhere the peak occurrence was reported in summer months in Indonesia and Karachi, Pakistan.^14^^,^^15^ Consumption of commercially prepared ice, ice creams and locally-made chilled drinks have been identified as some of major risk factors for typhoid fever in children in Karachi in summer season as reported by Luby et al, in addition to flooding that lead to the mixing of sewage and drinking water.^19^ Both factors might be the reasons for the highest prevalence of typhoid fever in summer.

On sex basis insignificant differences were observed, as it is equally present in both male and female children. This is in accordance with the studies conducted by Abdel Wahab et al, whereas Mubeena et al, and Fazil et al, found that males were infected predominantly as compared to females.^20^^-^^22^

## CONCLUSION

Typhoid fever is endemic in Quetta, Pakistan, occurring in both sexes equally under 15 years of age. These findings support the decision to vaccinate children against typhoid fever. A combination of mass vaccination and improvement in sanitation and water supply systems has been suggested as a method to control epidemics of typhoid fever in the high risk areas.

## Authors Contribution

***Muhammad Naeem Khan: ***Data collection and Research work of the study.

***Muhammad Shafee: ***Designing study and manuscript writing.

***Muhammad Arif Awan: ***Participated in subject selection and actively helped in selection of the patients.

***Abdul Samad: ***Editing and Final proof reading of the manuscript.

***Abdul Manan: ***Statistical Analysis of the study.

***Kamran Hussain:*** Contributed in laboratory Work.

***Abdul Wadood: ***Actively involved in sample collection and manuscript writing.
